# Distinct Effects of Estrogen on Mouse Maternal Behavior: The Contribution of Estrogen Synthesis in the Brain

**DOI:** 10.1371/journal.pone.0150728

**Published:** 2016-03-23

**Authors:** Gen Murakami

**Affiliations:** 1 Laboratory of Neurobiology and Behavior, The Rockefeller University, 1230 York Avenue, Box 275, New York, New York, 10065, United States of America; 2 Department of Psychology and Behavioral Neuroscience, Hamamatsu University School of Medicine, 1-20-1 Handayama, Higashi-ku, Hamamatsu, 431–3192, Japan; 3 Department of Regenerative and Infectious Pathology, Hamamatsu University School of Medicine, 1-20-1 Handayama, Higashi-ku, Hamamatsu, 431–3192, Japan; University of Rennes-1, FRANCE

## Abstract

Estrogen surge following progesterone withdrawal at parturition plays an important role in initiating maternal behavior in various rodent species. Systemic estrogen treatment shortens the latency to onset of maternal behavior in nulliparous female rats that have not experienced parturition. In contrast, nulliparous laboratory mice show rapid onset of maternal behavior without estrogen treatment, and the role of estrogen still remains unclear. Here the effect of systemic estrogen treatment (for 2 h, 1 day, 3 days, and 7 days) after progesterone withdrawal was examined on maternal behavior of C57BL/6 mice. This estrogen regimen led to different effects on nursing, pup retrieval, and nest building behaviors. Latency to nursing was shortened by estrogen treatment within 2 h. Moreover, pup retrieval and nest building were decreased. mRNA expression was also investigated for estrogen receptor α (ERα) and for genes involved in regulating maternal behavior, specifically, the oxytocin receptor (OTR) and vasopressin receptor in the medial amygdala (MeA) and medial preoptic area (MPOA). Estrogen treatment led to decreased ERα mRNA in both regions. Although OTR mRNA was increased in the MeA, OTR and vasopressin receptor mRNA were reduced in the MPOA, showing region-dependent transcription regulation. To determine the mechanisms for the actions of estrogen treatment, the contribution of estrogen synthesis in the brain was examined. Blockade of estrogen synthesis in the brain by systemic letrozole treatment in ovariectomized mice interfered with pup retrieval and nest building but not nursing behavior, indicating different contributions of estrogen synthesis to maternal behavior. Furthermore, letrozole treatment led to an increase in ERα mRNA in the MeA but not in the MPOA, suggesting that involvement of estrogen synthesis is brain region dependent. Altogether, these results suggest that region-dependent estrogen synthesis leads to differential transcriptional activation due to exogenous estrogen treatment, and thereby results in different effects on maternal behavior.

## Introduction

Estrogen plays an important role in initiation of maternal behavior in rodents [1]. In the first stages of pregnancy, progesterone levels increase and then decrease towards parturition [2]. At the same time as this progesterone withdrawal, plasma estrogen concentration increases, and this surge shortens the latency to onset of maternal behavior, which is known as sensitization. Additionally, the dominant effect of progesterone enhances the effect of estrogen regardless of treatment duration [3]. Hormone treatment that mimics this hormonal event at parturition (i.e., estrogen surge combined with progesterone withdrawal) also shortens the latency to sensitization in nulliparous females that have not experienced parturition [4]. In nulliparous female rats, it takes approximately 1 week to initiate maternal behavior without any treatment. However, hormone treatment shortens the latency to a few days, demonstrating the role of hormonal involvement at parturition in initiation of maternal behavior.

In rats, estrogen contributes to both reduced anxiety and increased pup approaches, both of which underlie the onset of maternal behavior [5, 6]. Nulliparous female rats treated with estrogen move more rapidly into a novel field compared with vehicle-treated rats [4]. In contrast, estrogen surge at parturition contributes to pup-reinforced lever pressing, indicative of a high motivational state towards pups [7]. Thus, these results suggest that estrogen regulates maternal behavior by modulating anxiety and approach to pups.

In comparison with rat studies, research on the effect of estrogen treatment on maternal behavior in mice is more controversial. Maternal behavior is severely impaired in estrogen receptor α (ERα) knockout mice and gonadally intact female mice with decreased ERα expression, due to shRNA treatment in the medial preoptic area (MPOA), showing the indispensable role of ERα in mouse maternal behavior [8, 9]. Moreover, estrogen treatment leads to a shortened latency to pup retrieval and recognition of ultrasonic vocalization, which contributes to pup recognition in ovariectomized (OVXed) female mice [10]. In contrast, other studies have shown that estrogen treatment in adult nulliparous female mice causes loss of maternal behavior and infanticidal behavior [11, 12]. In intact female mice, estrogen treatment alone results in poor nest-building ability [13, 14]. Furthermore, a recent study reported that gonadal hormones have no effect on crouching nor pup retrieval [15]. These results suggest that estrogen has complex effects on maternal behavior in mice.

This complexity may arise from estrogen synthesis in the brain. Estrogen at parturition is synthesized in the ovaries and secreted to the blood, with resulting effects in the brain [2]. However, estrogen is also synthesized in the brain [16, 17]. Given that virgin female mice spontaneously elicit rapid onset of maternal behavior without estrogen treatment [18], it is possible that brain-derived estrogen contributes to onset of maternal behavior in mice, regardless of ovarian estrogen surge at parturition. To determine the role of estrogen in mouse maternal behavior, we first treated OVXed nulliparous C57BL/6 mice with exogenous estrogen following progesterone withdrawal, thereby mimicking the hormonal event at parturition. Then, we analyzed maternal behavior such as nursing, pup retrieval, and nest building. Additionally, gene expression was examined in brain regions involved in regulation of maternal behavior, including the MPOA and medial amygdala (MeA). Finally, we addressed the contribution of brain-derived estrogen to maternal behavior by inhibiting estrogen synthesis in the brain of OVXed mice.

## Materials and Methods

### Animals

Inbred female C57BL/6 mice (aged 8–10 weeks) were housed singly under a reversed light–dark cycle (12:12 h, lights off at 10 am). Food and water were available *ad libitum*. Behavioral tests were performed within the first 4 h of the dark period, and videotaped for further analysis. All procedures were approved by The Rockefeller University Institutional Animal Care and Use Committee. The Public Health Services Policy on Humane Care and Use of Laboratory Animals was followed.

### Surgery and preparation of hormone containing capsules

Virgin female mice were OVXed under nembutal anesthesia (50 mg/kg body weight). Hormone treatments were performed by subcutaneous implantation of silastic capsules containing sesame oil (vehicle), 50 mg/mL progesterone (Sigma, MO, USA), or 0.5 mg/mL 17-β estradiol 3-benzoate (EB; Sigma), which delivered physiological concentration of these hormones, as demonstrated in previous studies [19]. Each capsule was made of 2-cm-long silastic tubing (ID: 0.078 inches, and OD: 0.125 inches), which was sealed off with medical grade silicone adhesive. To ensure constant hormone release from the capsules, prior to implantation all capsules were incubated in saline at 37°C overnight.

### Hormone treatment and behavioral analyses

#### Paradigm 1: maternal behavior analysis with unfamiliar pups

**Fig 1:**
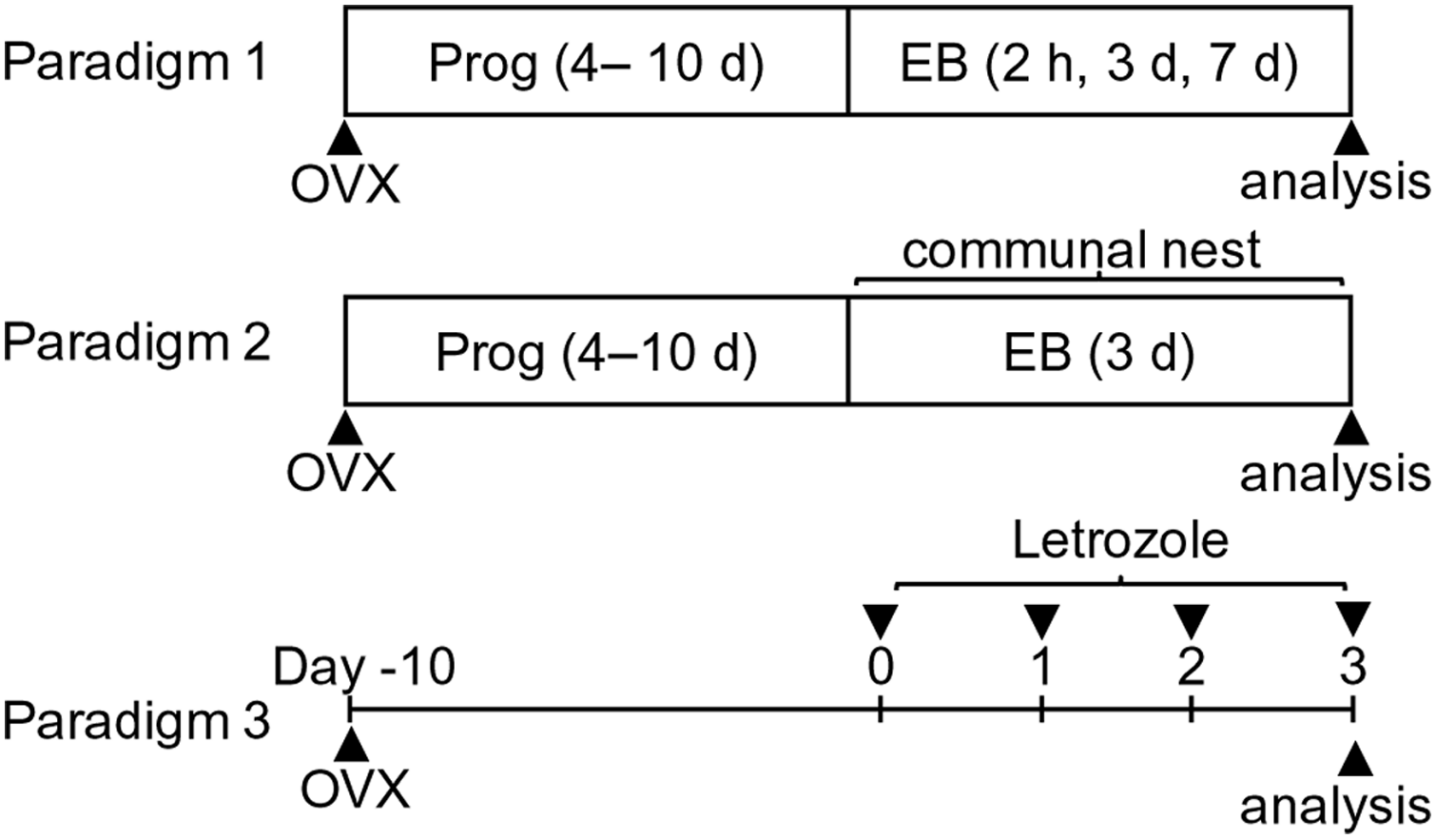
Schematic representation of the treatment paradigms. Maternal behavior was independently examined after hormonal treatments according to three paradigms. Paradigm 1: after OVX surgery, a progesterone capsule was implanted for 4–10 days. Simultaneously upon removal of the progesterone capsule, EB- or vehicle-containing capsules were implanted for 2 h, 3 days, or 7 days. Maternal behavior was then analyzed. Paradigm 2: hormonal treatment was the same as for 3 days EB treatment in paradigm 1. The difference from paradigm 1 was the use of familiar pups from communal nests for behavioral analyses. Paradigm 3: ten days after OVX surgery, mice were intraperitoneally injected with letrozole for 4 days. Four hours after the final treatment, maternal behavior was analyzed.

A total of 59 virgin female mice were OVXed and progesterone-containing capsules implanted. The mice were kept in their home cages for 4 to 10 days, and then the progesterone capsule removed and EB- or vehicle-containing capsules implanted. Mice were kept in their home cages for 2 h, 3 days, or 7 days prior to behavioral analysis (Fig 1, upper). For the behavioral analysis, all observations were started 10 min after placing the cage under the camera. Three pups (aged 2–4 postnatal days) from a primiparous mother were separately put in different corners of the cage. Latency to nursing and duration of nursing were measured for the first 15 min. Initiation of nursing was defined as the experimental mouse hovering over at least two pups to enable them to access the female’s ventral surface for more than 30 sec. If the mother did not show any sign of nursing behavior, the latency was counted for 15 min and then the pups placed in one corner by an experimenter before starting the next session.

For the next 30 min, nest materials were scattered within the cage, and latency to nest building and nest quality at the end of the session analyzed according to previously reported criteria [20]. Briefly, nest quality was rated as follows: score of 1 (poor), shavings evenly spread over the cage floor in the absence of an apparent nest area; 2 (fair), shavings evenly distributed over the cage floor and presence of a nest with a low wall; 3 (good), the majority of the shavings in one quadrant of the home cage and a nest with a high wall; and 4 (excellent), all shavings incorporated into a nest with a high wall. If a nest was not built, the latency was counted for 30 min and the nest materials placed in a corner with the pups before starting the next session.

In the final session, two pups were removed from the nest and separately repositioned to corners away from the nest. Latency to retrieval of both pups and the number of retrieved pups were measured for 15 min. If the experimental mouse did not retrieve both pups, the latency was counted for 15 min.

Using the same paradigm of EB treatment for 3 days, a total of 15 virgin female mice were examined for anxiety using the dark and light apparatus. An enclosed black acrylic box (40.0 × 20.5 × 20.5 cm) was set in the right half of an acrylic chamber (40.5 × 40.5 × 20.5 cm). Three pups were placed in the open compartment, which was directly illuminated by a 40 W white light placed above the compartment floor. Duration in the light compartment, number of entries, and latency before entering the compartment were scored for 5 min.

#### Paradigm 2: nursing behavior analysis with familiar pups from communal nesting

As in paradigm 1, a total of 54 virgin female mice were OVXed and prepared for vehicle and EB groups. EB treatment was performed for only 3 days. The main difference from paradigm 1 was the use of familiar instead of unfamiliar pups. For the familiar pups, mice were kept with a pregnant female mouse during progesterone treatment. On the day of parturition, experimental mice were subjected to EB treatment and kept for 3 days with the original postpartum female and her pups, resulting in communal nesting (Fig 1, middle). Nursing behavior was then analyzed in the same cage with three pups from the communal nest by removing the mother and extra pups. Latency to nursing was analyzed, as in paradigm 1. However, the amount of nursing was determined using a modified approach for the following reason. In paradigm 1, latency to nursing and its duration were analyzed for a total of 15 min. Therefore, mice showing early onset have a longer time for nursing, which can overestimate the actual amount of nursing. Thus, in paradigm 2, the observations started 10 min after initiation, and a time-sampling procedure performed, whereby nursing every 15 sec for 45 min was recorded.

Approach analysis was measured after 2 h and 3 days of EB treatment, as described in Litvin *et al*., with a few modifications [21]. Briefly, three pups from the communal nest were placed at the edge of a box (50 cm × 12 cm × 50 cm; constructed of white Plexiglas except for the front). Next, the experimental mouse was transferred to the opposite edge and the behavior was observed for 5 min. The box was divided into three equal sections and duration in the section with pups was analyzed.

#### Paradigm 3: maternal behavior analysis in mice treated with letrozole

A total of 15 virgin female mice were OVXed. Letrozole was prepared by dissolving in ethanol (8 mg/mL) followed by addition of 0.9% NaCl to a final concentration of 0.8 mg/mL. Ten days later, the mice were intraperitoneally injected with letrozole (Novartis, Basel, Switzerland) at a dosage of 4 μg/g body weight, or vehicle as a control once per day for 4 days (Fig 1, lower). Four hours after the final letrozole treatment, behavior was analyzed as described in paradigm 1. Additionally, approach to pups was analyzed as in paradigm 2.

### Molecular analysis

Molecular analysis was performed as described previously [22]. Briefly, following the behavioral studies, animals were deeply anesthetized with isoflurane and decapitated. The brains were quickly removed and stored at −80°C. Coronal slices of the entire brain were cut at 1-mm thickness using a mouse brain matrix, and the MPOA and MeA dissected using razor blades. Total RNA was isolated from each brain region using Trizol reagent, according to the manufacturer’s specifications (Invitrogen, San Diego, CA, USA). The amount and quality of total RNA were determined using a Nanodrop spectrophotometer (Thermo Scientific, Rockford, IL, USA).

Total RNA (0.5 μg) from each sample was reverse transcribed using a High-Capacity cDNA Archive Kit (Applied Biosystems, Foster City, CA, USA), according to the manufacturer’s specifications. The reverse transcription reaction consisted of 10 min at 25°C followed by 2 h at 37°C. Samples were diluted with nuclease-free H_2_O and stored at −80°C until use.

Quantitative real-time PCR (Q-PCR) was performed for the following gene products: ERα, oxytocin receptor (OTR), vasopressin receptor (V1aR) and 18S rRNA mRNA levels. Gene expression assays were purchased from Applied Biosystems for each gene product: ERα, Assay ID Mm00433149_m1; OTR, Assay ID Mm01182684_m1; V1aR, Assay ID Mm00444092_m1; and 18S rRNA, Assay ID 4352930E. Q-PCR reactions were performed using the TaqMan detection system (Prism 7000; Applied Biosystems) with the following conditions: 2 min at 50°C, 10 min at 95°C, and 40 cycles of 95°C for 15 sec and 60°C for 1 min. All Q-PCR reactions were run in triplicate, and relative gene expression levels calculated by delta Ct (dCt), subtracting the average cycle threshold (Ct) value for each gene product by the average Ct for 18S rRNA mRNA. The amount of each gene product in each brain region was set at 1 in the vehicle group using the following formula: *F*(*x*) = 2^-(X-Y)^. *X* is dCT for the gene of interest, and *Y* the average dCT from a comparable vehicle group.

### Statistical analysis

For all analyses, a minimum significance level of *P* = 0.05 was used. For behavioral analysis, two-tailed Student’s *t*-test was used for two group comparisons. For analysis of more than two groups, one-way ANOVA was used followed by Newman–Keuls multiple comparison test. For mRNA expression, dCt values were compared using the two-tailed Student’s *t*-test. For correlation analysis, the following equation: *t*_*0*_ *= r/sqrt[(1-r*^*2*^*)/ (N-2)]* was used. In this equation, *r* represents the correlation coefficient, *N* the number of samples, and then *t*_*0*_ distributes as *t* with *d*.*f*. = *N*-2.

## Results

### The effect of estrogen on maternal behavior with unfamiliar pups (paradigm 1)

To examine the effect of estrogen on maternal behavior, we examined nursing, pup retrieval, and nest building behaviors in OVXed virgin female C57BL/6 mice that had experienced progesterone withdrawal followed by EB or vehicle treatment for 2 h, 3 days, and 7 days (Fig 1, paradigm 1). A total of 59 mice were used and divided into these four groups. In this paradigm, unfamiliar pups from host mothers were used to observe maternal behavior in the home cage of the experimental mouse. No differences were observed among mice treated with vehicle for different durations in all behavioral analyses, therefore these mice were combined into a single vehicle group.

**Fig 2:**
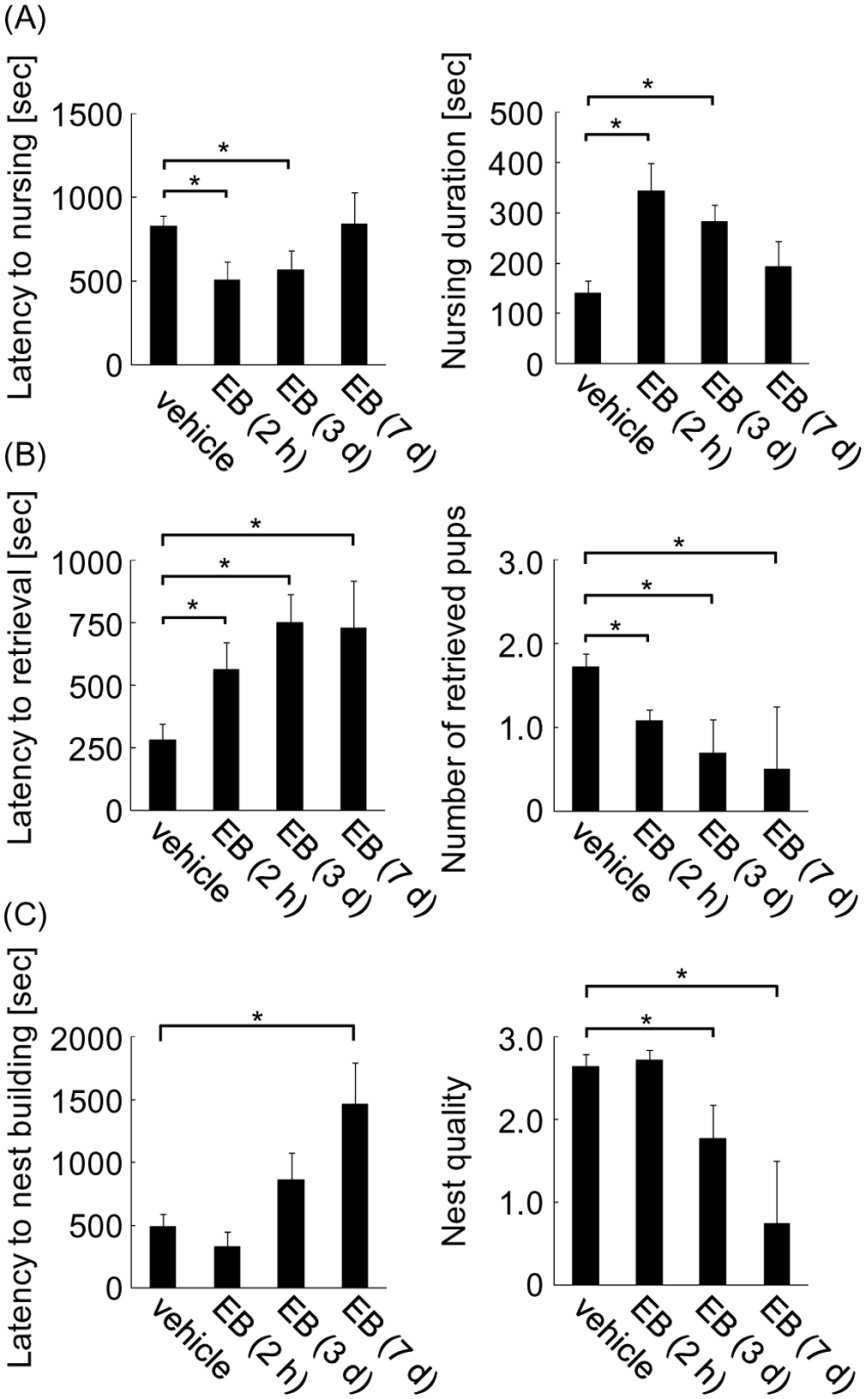
Maternal behavior of OVXed mice after estrogen treatment. Maternal behavior of OVXed mice was analyzed using unfamiliar pups after progesterone withdrawal followed by EB treatment for 0 (vehicle), 2 h, 3 days, and 7 days. (A) Latency to nursing (left) and duration of nursing (right) were analyzed. (B) Latency to pup retrieval (left) and number of retrieved pups (right) were analyzed. (C) Latency to nest building (left) and nest quality (right) were analyzed.

Mice treated with EB for 2 h exhibited significantly shortened latencies to nursing. However, longer EB treatment gradually increased the latency, and at 7 days of treatment it was comparable to vehicle-treated mice (Fig 2A, left; one-way ANOVA, *F*_(3, 37)_ = 4.90, *P < 0.05). Nursing duration was concordant with the change in latency. Mice treated with EB for 2 h showed longer nursing duration, while extended EB treatment for 7 days reduced the duration to a similar level as vehicle-treated mice (Fig 2A, right; one-way ANOVA, *F*_(3, 41)_ = 6.54, *P < 0.05). These results show that EB has a dual effect on nursing: a rapid improving effect within 2 h, followed by a slow inhibitory effect for up to 7 days.

In contrast to nursing, estrogen only exerted an inhibitory effect on pup retrieval. Compared with vehicle-treated mice, onset of pup retrieval was delayed by 2-h EB treatment. This delay was further exacerbated by 3 days of treatment, and persisted until 7 days (Fig 2B, left; one-way ANOVA, *F*_(3, 52)_ = 7.23, *P < 0.05). Accompanying this increased latency, fewer pups were retrieved by mice treated with EB for 2 h, and the number was even lower for animals treated with EB for 3 and 7 days (Fig 2B, right; one-way ANOVA, *F*_(3, 53)_ = 7.23, *P < 0.05). Similar to retrieval behavior, nest building behavior was impaired by EB treatment. Latency to nest building increased (Fig 2C, left; one-way ANOVA, *F*_(3, 54)_ = 4.58, *P < 0.05) and nest quality decreased at 3 and 7 days of EB treatment (Fig 2C, right; one-way ANOVA, *F*_(3, 55)_ = 6.14, *P < 0.05). These results indicate that only inhibitory actions on pup retrieval and nest building behaviors are observed following EB treatment.

### The effect of estrogen on anxiety and approach to pups

**Fig 3:**
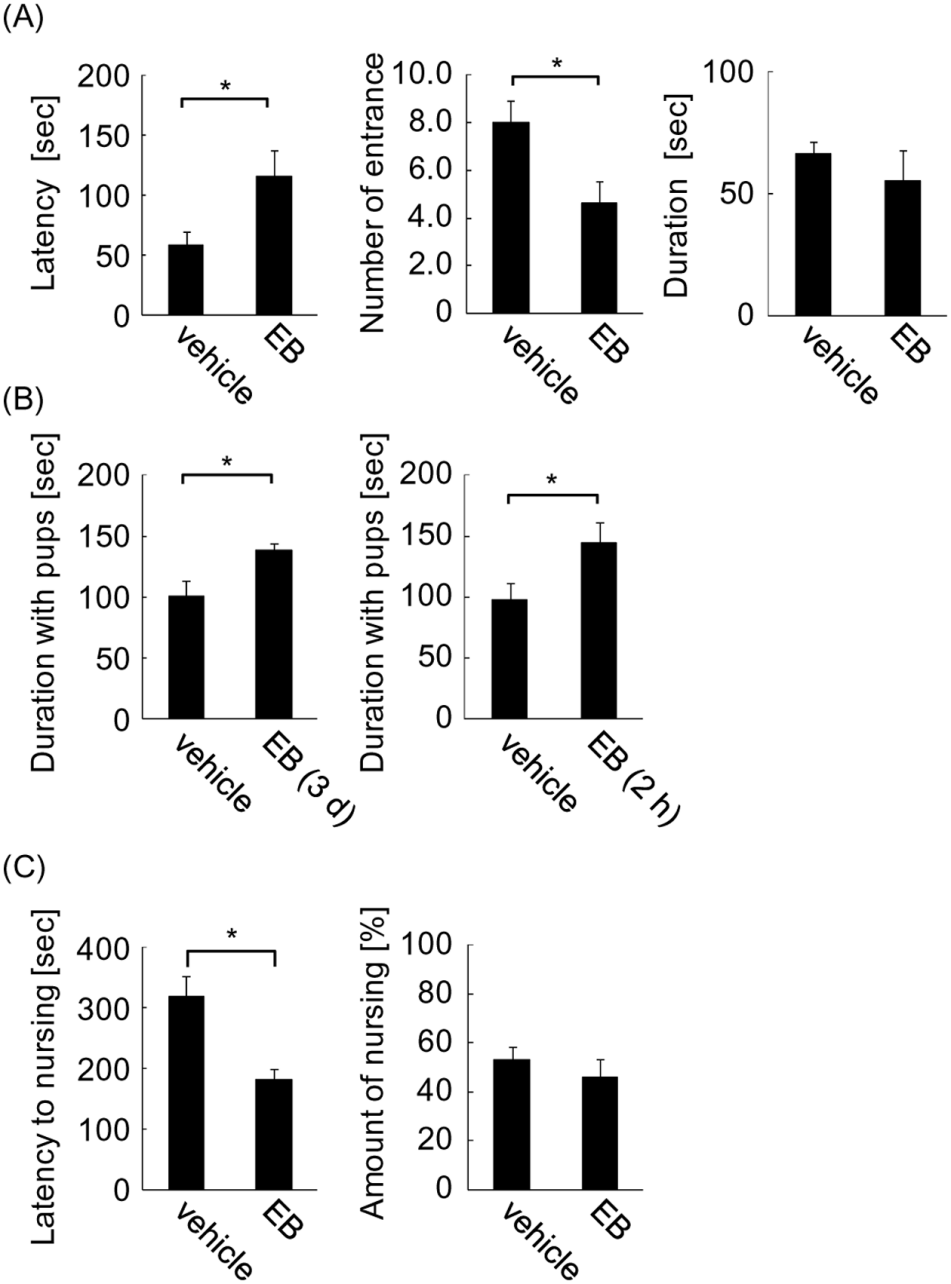
Effect of estrogen on anxiety and approach to pups. (A) Anxiety of OVXed mice following progesterone withdrawal and 3 days EB treatment, was measured using the light/dark apparatus with unfamiliar pups placed in the light compartment. Latency to entering the light compartment (left), number of entries (middle), and duration in the light compartment (right) were examined. (B) Approach to familiar pups from communal nests was examined in OVXed mice treated with EB for 3 days (left) or 2 h (right). (C) Latency to nursing (left) and amount of nursing (measured by the time-sampling procedure) (right) were analyzed using familiar pups from communal nests in OVXed mice treated with EB for 3 days.

Anxiolytic and prosocial effects are thought to be primitive roots of maternal behavior, with estrogen inducing these effects in rats [4, 7]. To examine these effects in mice, anxiety and approach to pups were analyzed after progesterone withdrawal followed by EB treatment or vehicle in OVXed mice. Only 3 days of EB treatment was examined because of the behavioral effect observed in paradigm 1. The effect of estrogen on anxiety was analyzed using the light/dark apparatus with pups placed in the light compartment. This environment creates a more stressful situation than in the home cage, and is expected to exaggerate the anxiolytic effect of estrogen. Anxiety was determined by the latency to enter the light compartment, number of entrances, and duration in the light compartment. A total of 15 mice were used and divided into vehicle and EB groups. In contrast to previous studies demonstrating an anxiolytic effect of EB on rats [4], EB-treated mice showed increased anxiety, as evidenced by longer latencies and decreased number of entrances into the light compartment, compared with vehicle mice (Fig 3A, left and middle; two-tailed Student’s *t*-test, *t* = 2.16 for latency; *t* = 2.53 for number, *P < 0.05). Although the duration in the light compartment was similar in EB- and vehicle-treated mice (Fig 3A, right; 67 sec for vehicle and 55 sec for EB), these results indicate that estrogen treatment enhances anxiety.

To examine the prosocial effect of estrogen, approach to pups was investigated using familiar pups from communal nesting. This decreases the anxiety of experimental mice to the pups, and is expected to reduce the anxiolytic effect of estrogen [23, 24]. After progesterone withdrawal, experimental mice spent 3 days with the pups in communal nests during EB or vehicle treatment (Fig 1, paradigm 2). For this experiment, a total of 12 mice were used and divided into two groups. Treatment of EB for 3 days increased pup approaches, as indicated by increased duration with pups (Fig 3B left; two-tailed Student’s *t*-test, *t* = 2.41, *P < 0.05). Because EB treatment as short as 2 h was sufficient to shorten the latency to nursing, the effect of 2-h EB treatment on pup approaches was also investigated. This short treatment increased the duration time with the pups, as seen after 3 days EB treatment (Fig 3B, right; two-tailed Student’s *t*-test, *t* = 2.75, *P < 0.05), suggesting that estrogen rapidly increases pup approaches within 2 h.

Using familiar pups from communal nests, nursing behavior was also examined. A total of 54 mice were used and divided into vehicle and EB groups. Three days of EB treatment shortened the latency to nursing (the same as in the analysis with unfamiliar pups) (Fig 3C, left; two-tailed Student’s *t*-test, *t* = 2.47, *P < 0.05). To exclude the effect of different latencies, the amount of nursing was analyzed 10 min after initiation using a time-sampling procedure for 45 min. This observation approach found no effect of estrogen on the amount of nursing (Fig 3C, right). Overall, these results suggest that estrogen shortens the latency, but not the amount of nursing once initiated. It should be noted that latency to nursing with familiar pups was much shorter than with unfamiliar pups (Fig 2A, left), showing reduced anxiety of the experimental mice to familiar pups from communal nests. However, reduced anxiety did not change the effect of EB treatment on nursing. These results suggest that the observed estrogen-induced shortened latency to nursing depends on increased pup approaches.

### The effect of estrogen on expression of ERα, OTR, and V1aR

**Fig 4:**
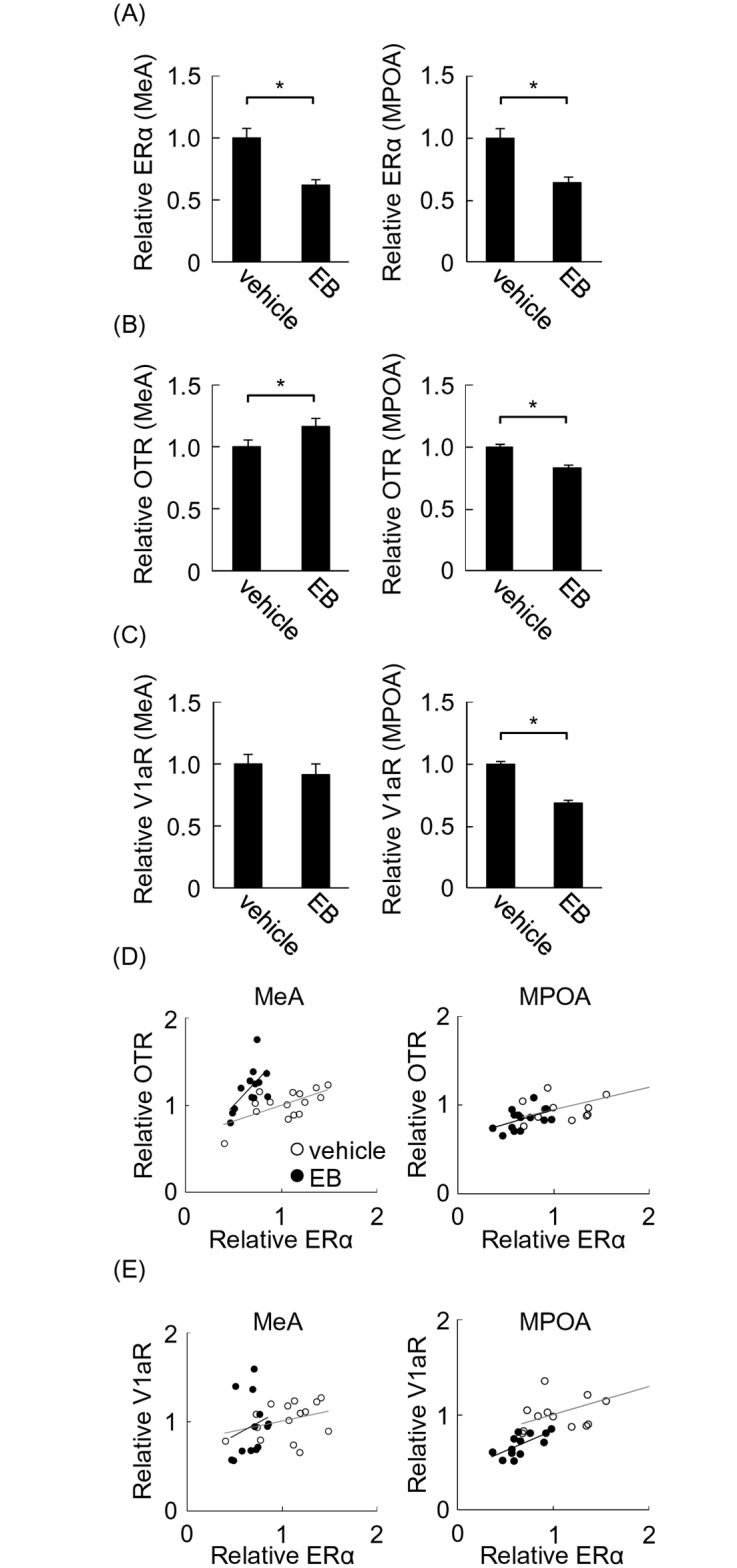
mRNA expression in OVXed mice after 3 days-EB treatment following progesterone withdrawal. (A) Estrogen receptor α (ERα) mRNA in the medial amygdala (MeA) and medial preoptic area (MPOA) was examined. (B) Oxytocin receptor (OTR) mRNA in the MeA and MPOA was examined. (C) Vasopressin receptor V1a (V1aR) mRNA in the MeA and MPOA was examined. (D) Correlation between ERα and OTR mRNA in the MeA and MPOA. (E) Correlation between ERα and V1aR in the MeA and MPOA. Quantity of mRNA was determined by the relative ratio against the vehicle group.

To gain insight into the molecular mechanisms of how estrogen differently regulates maternal behavior, ERα mRNA expression levels were determined in brain regions involved in regulation of maternal behavior, such as the MeA and MPOA of the hypothalamus. A total of 15 mice were randomly chosen for each group from mice that were examined for nursing with familiar pups (paradigm 2). Mice were sacrificed immediately after behavioral analysis. In both the MeA and MPOA, EB treatment led to an approximately 60% reduction in ERα mRNA compared with the vehicle group (Fig 4A; two-tailed Student’s *t*-test, *t* = 3.79 for MeA, *t* = 3.86 for MPOA, *P < 0.05). This suggests negative autoregulation of ERα by estrogen, consistent with previous reports [25, 26]. Next, OTR and V1aR mRNA levels were examined because estrogen binding to ERα activates expression of these genes. Moreover, these receptors are crucial to the onset of maternal behavior [27, 28]. In the MeA, EB treatment increased OTR mRNA to 117% compared with the vehicle group, whereas it decreased to 83% in the MPOA (Fig 4B; *t* = 2.37 for MeA, *t* = 2.43 for MPOA, *P < 0.05). Although V1aR mRNA levels were comparable in the MeA of EB- and vehicle-treated mice (Fig 4C, left), they reduced to 69% in the MPOA of EB-treated mice (Fig 4C, right; two-tailed Student’s *t*-test, *t* = 4.31, *P < 0.05).

To further investigate how estrogen differentially regulates OTR and V1aR mRNA through ERα, correlations between ERα and OTR or V1aR mRNAs were examined. Positive correlations were observed between ERα and OTR mRNA in the MeA in vehicle- and EB-treated groups (Fig 4D, left; *r* = 0.72, *t* = 3.91, *P* < 0.01 for vehicle; *r* = 0.71, *t* = 3.66, *P* < 0.01 for EB). Thus, OTR expression is under estrogen control via ERα in both groups. However, in the EB group, the regression line has a larger slope compared with the vehicle group (0.37 for vehicle and 1.21 for EB). Because the slope of the regression line represents the ratio of OTR to ERα mRNA, a larger slope shows activated OTR transcription in the EB treatment group. Therefore, despite the reduction of ERα in the MeA, more transcriptional activation of OTR results in a net increase of OTR mRNA compared with vehicle mice. In the MPOA, OTR and ERα mRNA were correlated in both the vehicle and EB groups (Fig 4D, right; *r* = 0.67, *t* = 3.19, *P* < 0.01 for vehicle; *r* = 0.52, *t* = 2.21, *P* < 0.05 for EB). The slopes in both groups were practically identical (0.26 for vehicle and 0.33 for EB), showing that EB treatment did not activate OTR transcription in the MPOA. Decreased ERα expression combined with lack of transcriptional activation results in decreased OTR mRNA in the MPOA.

Similar to OTR, V1aR positively correlated with ERα in the MPOA of both vehicle and EB groups (Fig 4E, right; *r* = 0.68, *t* = 3.27, *P* < 0.01 for vehicle; *r* = 0.67, *t* = 3.20, *P* < 0.01 for EB). The regression line slopes were almost the same in both groups (0.45 for vehicle and 0.30 for EB), showing no transcriptional activation by EB treatment. In the MeA, V1aR did not correlate with ERα mRNA in either group (Fig 4E, left; *r* = 0.32, *t* = 1.24, *P* = 0.24 for vehicle; *r* = 0.22, *t* = 0.77, *P* = 0.46 for EB). It should be noted that V1aR mRNA expression levels in the MeA were only 10% that observed in the MPOA, and 1% that in the MeA of male mice (by estimation from qPCR analysis [22]). Given the much lower expression, it is predicted that in the MeA, the contribution of V1aR in female mice is much smaller. Taken together, the results demonstrate that estrogen treatment activates OTR transcription in the MeA. However, neither OTR nor V1aR expression are activated in the MPOA, suggesting region-dependent activation of gene expression.

### The effect of letrozole on maternal behavior (paradigm 3)

**Fig 5:**
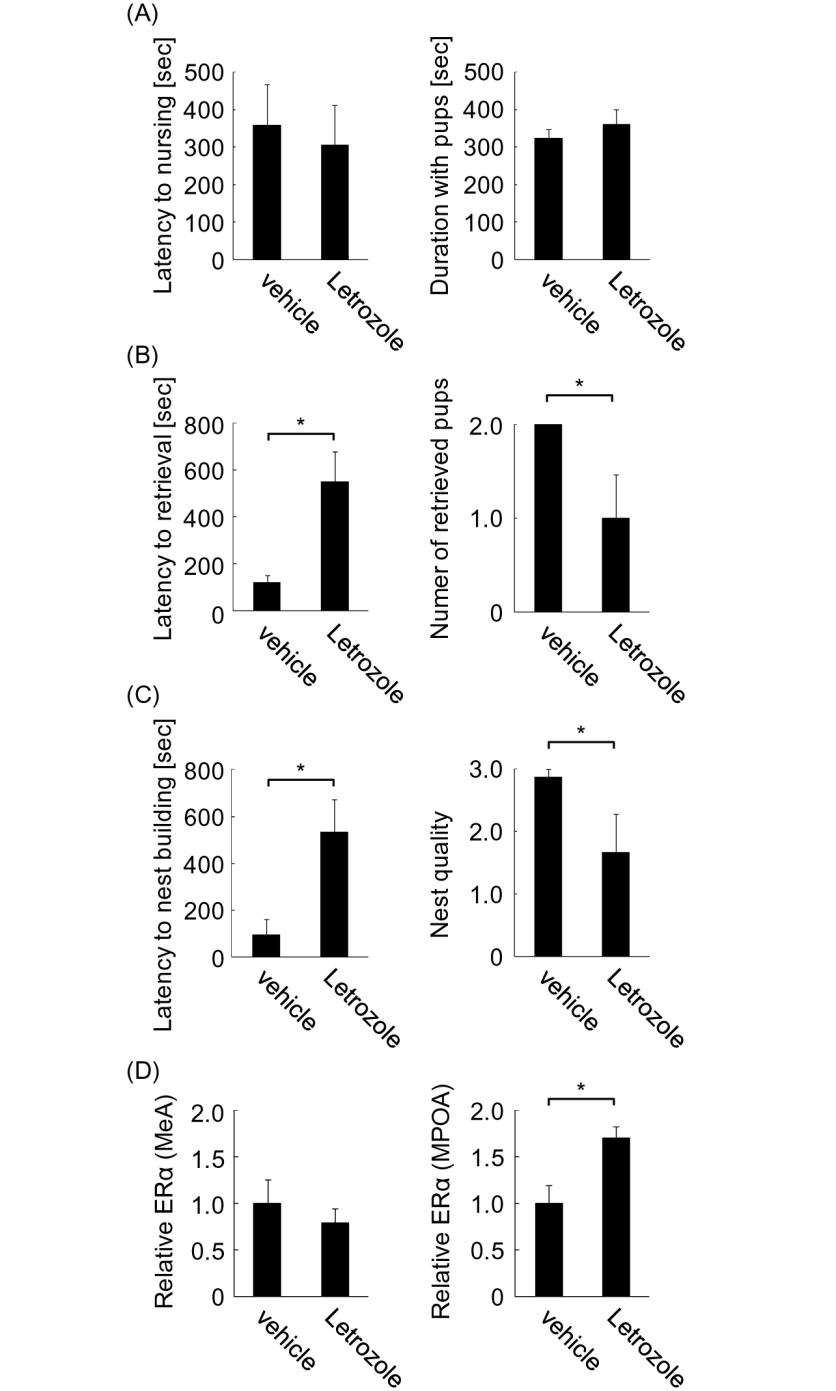
Maternal behavior of OVXed mice treated with letrozole. Maternal behavior of OVXed mice was analyzed using unfamiliar pups after treatment with or without letrozole. (A) Latency to nursing (left) and approach to pups (right) were analyzed. (B) Latency to pup retrieval (left) and number of retrieved pups (right) were analyzed. (C) Latency to nest building (left) and nest quality (right) were analyzed. (D) ERα mRNA was analyzed in the medial amygdala (MeA) (left) and medial preoptic area (MPOA) (right) of mice treated with or without letrozole immediately after behavioral analysis. Quantity of mRNA was determined by the relative ratio against vehicle group.

Estrogen synthesis in the brain may contribute to region-dependent activation of transcription by estrogen treatment [16, 17], because the effect of exogenous estrogen can be modified in brain regions where estrogen is synthesized. To determine the contribution of estrogen synthesis in the brain to maternal behavior, latency to nursing, pup retrieval, nest building, and approach to unfamiliar pups were investigated after systemic treatment of OVXed virgin female mice with letrozole, an inhibitor of estrogen synthesis, or vehicle for 4 days (Fig 1, paradigm 3). This treatment paradigm is enough to reduce estrogen synthesis in the brain [29–31]. A total of 18 mice were used and divided into these two groups. Letrozole treatment had no effect on latency to nursing (Fig 5A, left). Additionally, approach to pups was not affected (Fig 5A, right). In contrast, letrozole treatment impaired pup retrieval behavior, as shown by significantly delayed retrieval onset (Fig 5B, left; two-tailed Student’s *t*-test, *t* = 3.27, *P < 0.05) and reduced average number of retrieved pups (Fig 5B, right; two-tailed Student’s *t*-test, *t* = 2.62, *P < 0.05). Similarly, letrozole significantly delayed initiation of nest building (Fig 5C, left; two-tailed Student’s *t*-test, *t* = 3.03, *P < 0.05). Nest quality was also decreased in the letrozole group (Fig 5C, right; two-tailed Student’s *t*-test, *t* = 2.22, *P < 0.05). These results show a contribution of estrogen synthesis in the brain to pup retrieval and nest building, but not to nursing and approach to pups.

Because ERα expression is negatively autoregulated by estrogen (Fig 4A), it is possible that brain-synthesized estrogen also contributes to its regulation. To investigate this possibility, ERα mRNA was examined in the brains of mice treated with letrozole in the above experiment. Letrozole treatment enhanced expression of ERα mRNA to 170% that of the vehicle group in the MPOA but not the MeA (Fig 5D; two-tailed Student’s *t*-test, *t* = 2.37, *P < 0.05 for MPOA). These results suggest the possible involvement of estrogen synthesis in the MPOA but not the MeA.

## Discussion

### Estrogen in mouse maternal behavior

This study shows that estrogen contributes to nursing, pup retrieval, and nest building in C57BL/6 mice in different fashions. Furthermore, it shows potent contribution of brain-synthesized estrogen to maternal behavior. Prior studies genetically manipulating the ERα gene have clearly shown that ERα is necessary for normal maternal behavior [8, 9]. In contrast, estrogen treatment results in ambiguous behavioral effects in mice. This study shows that region-dependent synthesis of estrogen in the brain may contribute to maternal behavior and explain these discrepancies.

With nursing behavior, estrogen shortened the latency but had no effect on the amount of nursing after its onset. This is consistent with previous findings demonstrating that estrogen is particularly important for initiation of maternal behavior but not its maintenance [15, 32, 33]. Upon detailed examination of the nursing analysis, I found that the effect of estrogen consisted of two phases: a rapid improving effect (within 2 h) followed by a slow inhibitory effect, which is explained by decreased ERα expression observed in the MPOA and MeA. Indeed, exogenous estrogen treatment reduces ERα expression in the MPOA of rats [34], indicating negative autoregulation of ERα expression by estrogen [25, 26]. A two-phased effect of estrogen is physiologically reasonable because the time window for inducing maternal behavior upon presentation of pups is limited in postpartum mothers, and if the mothers are separated from pups during this period, maternal behavior cannot be induced in various species [35, 36].

In contrast to nursing behavior, estrogen treatment had only an inhibitory effect, with no rapid improving effect, on pup retrieval and nest building. Poor nest building behavior after estrogen treatment has previously been reported in mice [13, 14]. The different effect of estrogen on retrieval and nest building, compared to nursing, indicates that different mechanisms for estrogen’s action underlie each behavior.

### Contribution of estrogen synthesis in the brain

**Fig 6:**
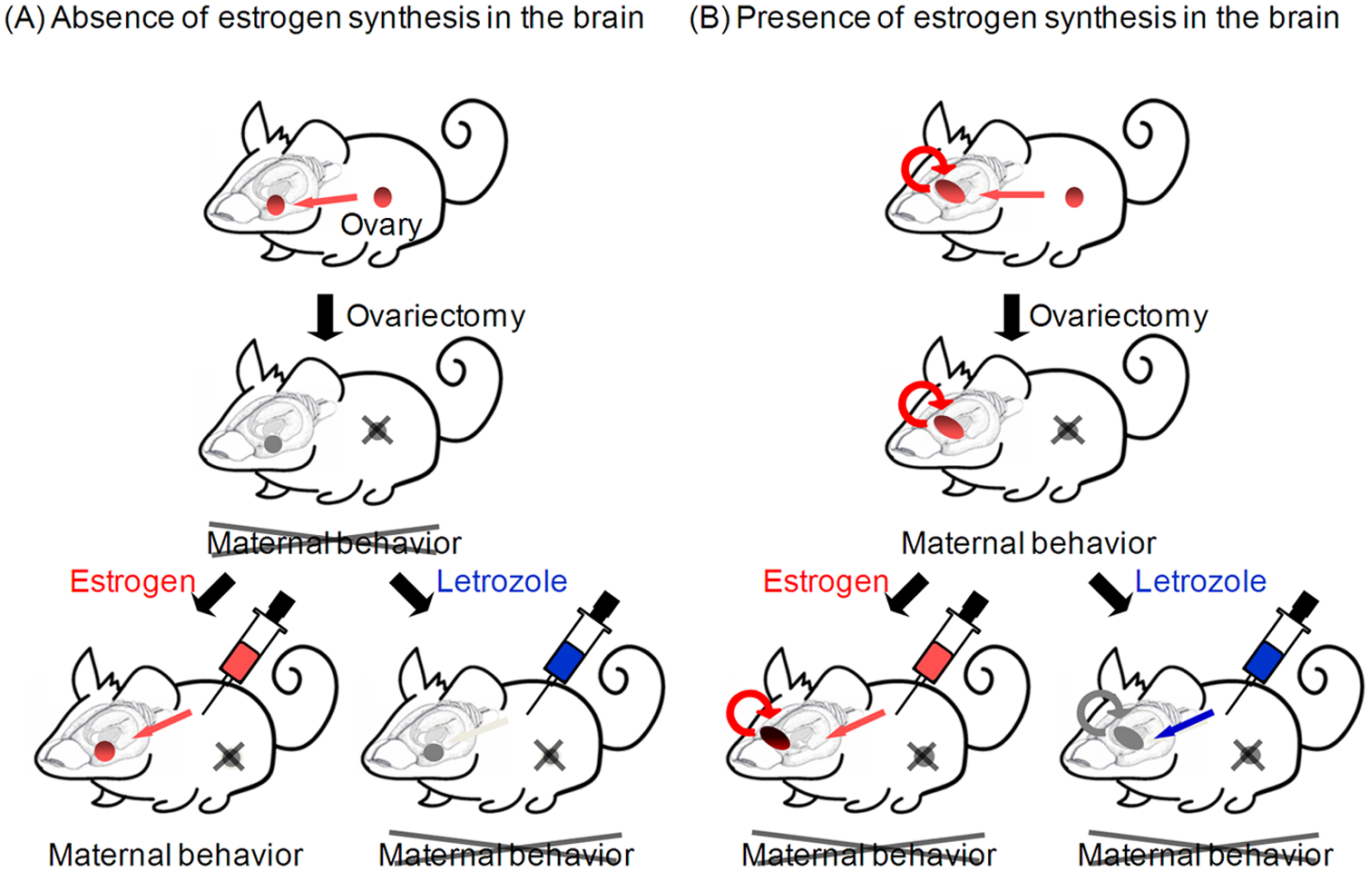
Schematic illustration of estrogen and letrozole contribution in OVXed mice. (A) In the absence of brain estrogen synthesis, estrogen treatment restores maternal behavior in OVXed mice, while letrozole has no effect. (B) In the presence of brain estrogen synthesis, further estrogen treatment leads to excess estrogen and impairs maternal behavior in OVXed mice. In contrast, letrozole treatment inhibits estrogen synthesis in the brain and impairs maternal behavior.

The lack of an improving effect of estrogen treatment on pup retrieval and nest building may appear to conflict with previous findings showing an indispensable role of ERα in the maternal behavior of mice [8, 9]. Estrogen synthesis in the brain may explain this discrepancy. When estrogen is synthesized, further treatment would have no improving effect and excess estrogen would have only a slow inhibitory effect [19] (Fig 6B). Indeed, this study found an inhibitory effect of letrozole on pup retrieval and nest building in OVXed mice, which indicates the contribution of estrogen synthesis to these behaviors. The MPOA is a predominant nucleus for governing pup retrieval and nest building behaviors. Studies with rats show that lesions of lateral MPOA projections severely disrupt pup retrieval and nest building, but only slightly disrupted nursing behavior. This indicates that the MPOA is particularly important for retrieval and nest building behaviors [37–39]. Furthermore, the MPOA is the most critical site for estrogen synthesis in other species [40]. Here, letrozole increased ERα mRNA expression in the MPOA, which may be due to withdrawal of endogenously synthesized estrogen under negative autoregulation. Because of estrogen synthesis in the MPOA, exogenous estrogen treatment would fail to activate transcription of OTR and V1aR expression, and excess estrogen would inhibit pup retrieval and nest building. Given that ERα in the MPOA is involved in regulation of anxiety [41], it is possible that excess estrogen in the MPOA increases anxiety, and this mechanism contributes to inhibition of these behaviors. Because the MPOA is also involved in other social behaviors such as sexual behavior and aggression towards intruders in a semi-natural environment [9], estrogen synthesis in the MPOA may also contribute to regulation of these behaviors.

In contrast, letrozole had no effect on latency to nursing, suggesting that nursing initiation is independent from estrogen synthesis in the brain. This implies that estrogen is not synthesized in brain regions dominantly governing nursing behavior. Furthermore, shortened latency to nursing by estrogen treatment may be due to lack of estrogen synthesis in these regions, because exogenous estrogen would only have an effect when the amount of estrogen is not sufficient (Fig 6A). The MeA may be a candidate nucleus contributing to the onset of nursing. The MeA receives direct projections from olfactory systems and sends projections to the MPOA [42–44]. Because ERα expression in the MeA is important for social interaction [22], it is possible that the same mechanism underlies the approach to pups and nursing behavior. Additionally, this study found no modification of ERα mRNA by letrozole or activation of OTR transcription by estrogen treatment in the MeA, consistent with the notion that estrogen synthesis is not dominant in this region. Altogether, these results support the idea that exogenous estrogen has an effect in brain regions important for the onset of nursing, where estrogen synthesis is not dominant, as in the MeA. It should be noted that in contrast to humans, the adrenal gland of rats and mice is devoid of P450 17α-hydroxylase, which is responsible for estrogen synthesis [45, 46], and OVX results in no plasma estrogen [47]. This means that letrozole treatment in OVXed mice does not have an effect in peripheral organs, only in the brain.

### Conclusion

In conclusion, this study shows the important role of estrogen in maternal behavior of C57BL/6 mice, which is the same as in other species. Exogenous estrogen shortens the latency to nursing within 2 h, and this effect is gradually eliminated along with treatment duration. Conversely, letrozole interferes with pup retrieval and nest building behaviors, suggesting that estrogen synthesis in brain regions such as the MPOA may contribute to these behaviors. Because these results are based on OVXed mice, it is important to assess the contribution of brain-derived estrogen to maternal behavior in intact female mice at parturition, during which various hormonal changes occur. Direct measurement of estrogen concentration in brain regions related to maternal behavior is also needed in future studies. Altogether, our findings provide a potential mechanism for the different actions of brain- and ovary-derived estrogen on mouse maternal behavior.

## Abbreviations

EB: estrogen benzoate
ERα: estrogen receptor α
OVX: ovariectomy
MPOA: medial preoptic area
MeA: medial amygdala
OTR: oxytocin receptor
V1aR: vasopressin receptor V1a
